# Qiliqiangxin Capsules Optimize Cardiac Metabolism Flexibility in Rats With Heart Failure After Myocardial Infarction

**DOI:** 10.3389/fphys.2020.00805

**Published:** 2020-07-17

**Authors:** Wenkun Cheng, Lei Wang, Tao Yang, Aiming Wu, Baofu Wang, Tong Li, Ziwen Lu, Jingjing Yang, Yang Li, Yangyang Jiang, Xiaoxiao Wu, Hui Meng, Mingjing Zhao

**Affiliations:** Key Laboratory of Chinese Internal Medicine of Ministry of Education and Beijing, Dongzhimen Hospital, Beijing University of Chinese Medicine, Beijing, China

**Keywords:** metabolic flexibility, heart failure, Qiliqiangxin capsules, metabolic modulation, border area, remote area

## Abstract

Metabolic modulation is a promising therapy for ischemic heart disease and heart failure. This study aimed to clarify the regional modulatory effect of Qiliqiangxin capsules (QLQX), a traditional Chinese medicine, on cardiac metabolic phenotypes. Sprague–Dawley rats underwent left anterior descending coronary artery ligation and were treated with QLQX and enalapril. Striking global left ventricular dysfunction and left ventricular remodeling were significantly improved by QLQX. In addition to the posterior wall, QLQX also had a unique beneficial effect on the anterior wall subject to a severe oxygen deficit. Cardiac tissues in the border and remote areas were separated for detection. QLQX enhanced the cardiac ^18^F-fluorodeoxyglucose uptake and the levels and translocation of glucose transport 4 (GLUT4) in the border area. Meanwhile, it also suppressed glucose transport 1 (GLUT1) in both areas, indicating that QLQX encouraged border myocytes to use more glucose in a GLUT4-dependent manner. It was inferred that QLQX promoted a shift from glucose oxidation to anaerobic glycolysis in the border area by the augmentation of phosphorylated pyruvate dehydrogenase, pyruvate dehydrogenase kinases 4, and lactic dehydrogenase A. QLQX also upregulated the protein expression of fatty acid translocase and carnitine palmitoyl transferase-1 in the remote area to possibly normalize fatty acid (FA) uptake and oxidation similar to that in healthy hearts. QLQX protected global viable cardiomyocytes and promoted metabolic flexibility by modulating metabolic proteins regionally, indicating its potential for driving the border myocardium into an anaerobic glycolytic pathway against hypoxia injuries and urging the remote myocardium to oxidize FA to maximize energy production.

## Introduction

Ischemic heart disease and chronic heart failure (HF) remain major causes of mortality worldwide despite advances made dramatically in therapeutic approaches on neurohormones and devices (Lytvyn et al., 2017). “Metabolic modulation” proved to be a promising pharmacological strategy by optimizing cardiac substrate preference given that substrate perturbations might underlie contractile dysfunction and left ventricular (LV) remodeling (Noordali et al., 2018; Birkenfeld et al., 2019). However, only few metabolic regulators are available in the clinic, accounting for insufficient evidence. Healthy adult hearts prefer fatty acids (FA) as the predominant energy source for 60–90% of adenosine triphophate (ATP) production (Kolwicz et al., 2013). Of importance is that “metabolic flexibility” empowers a normal heart to switch between FA and other alternative substrates, including glucose, ketone bodies, and amino acids, depending on alterations in physiological conditions, thus ensuring continuous contractility (Gupta and Houston, 2017; Bertero and Maack, 2018). It is generally considered that the failing heart prefers glucose over FA, eliciting a current therapeutic paradigm that increasing glucose oxidation (or depressing FA utilization) is cardioprotective as glucose expends less oxygen for ATP generation (Liao et al., 2002; Li et al., 2016). Unexpectedly, either increasing glucose uptake by specific overexpression of GLUT1 in the murine heart (Yan et al., 2009) or depriving FA acutely using acipimox (Wolf et al., 2016) deteriorates cardiac metabolic inflexibility and functional failure. In addition, high fat feeding to increase FA oxidation (FAO) improves LV function in rats with myocardial infarction (MI) (Berthiaume et al., 2012). Hence, whether to further promote glucose utilization or normalize substrate to FA still remains unresolved (Lionetti et al., 2011). Currently, agents that stimulate glucose or FA might be tested further as they induce inflexibility. It is cautiously concluded that promoting metabolic extremes, either preference or inhibition for any one substrate, makes the heart further lose its flexibility and become vulnerable to adverse injuries under stressors. FA and glucose metabolism are both indispensable, and recovering substrate flexibility is a more desirable target for long-term metabolic modulation.

Recently, region-dependent LV remodeling after MI invoked great interest for its potential to detect more sensitive variables for prognosis and treatment (Torres et al., 2018; van Duijvenboden et al., 2019). According to the proximal–distal axis, the infarcted myocardium is divided into an infarcted area, a border area (adjacent to scar; hypoperfused), and a remote area (distal to scar; perfused) (van Duijvenboden et al., 2019). A series of studies confined the border area to be a discrete zone with hallmarks of unique structural (Sharov et al., 1997), mechanical (Torres et al., 2018), and transcriptional properties (van Duijvenboden et al., 2019) distinct from the remote myocardium. Importantly, metabolic remodeling post-MI is also identified as spatial non-uniformity. The transcriptome and regulatory profiles of mitochondrial oxidative phosphorylation and FAO and glucose metabolism were more perturbed in the border area than in remote areas (Rosenblatt-Velin et al., 2001; van Duijvenboden et al., 2019). It was proposed that the regional modulation of cardiac substrate utilization, that is, selecting the appropriate ones for distinct areas based on their local microenvironment, is a promising target for motivating metabolic flexibility.

Qiliqiangxin capsules (QLQX), a listed traditional Chinese medicine comprising 11 herbs, were approved by the China Food and Drug Administration in 2004. Its main components include Astragali Radix, Ginseng Radix et Rhizoma, Aconiti Lateralis Radix Preparata, Salvia Miltiorrhiza Radix et Rhizoma, Alismatis Rhizoma, Descuraunia Semen, Cinnamomi Ramulus, Carthami Flos, Periplocae Cortex, Polygonati Odorati Rhizoma, and Citri Reticulatae Pericarpium (Li et al., 2013; Zhang et al., 2019). The favorable efficacy of QLQX in HF therapy has been confirmed by a multicentral large-scale clinical trial (Li et al., 2013; Tang and Huang, 2013) and several lab experiments (Tao et al., 2015; Liang et al., 2016; Han et al., 2018). Interestingly, QLQX has a multi-target enhancing effect on cardiac FAO, glucose oxidation, and glycolysis (Shen et al., 2017; Wang et al., 2017, 2018), implying that QLQX might modulate substrate utilization by promoting metabolic flexibility. Thus, this study was designed to ascertain whether QLQX enabled the border and remote myocardium to choose individual optimal metabolic phenotype for improving global energetic efficiency. Glucose utilization was observed by positron emission tomography (PET), and changes in a range of metabolic enzymes were detected in both border and remote areas post-MI.

## Materials and Methods

### HF Model After MI in Rats

Male Sprague–Dawley rats (230–250 g) were obtained from Beijing Vital River Laboratory Animal Technology Co., Ltd. [Animal license number: SCXK (Beijing) 2016-0006]. HF after MI was induced surgically in rats by left anterior descending coronary artery (LAD) ligation as described previously (Wu et al., 2013; Yang et al., 2019). Briefly, following anesthesia with sodium pentobarbital (40 mg/kg intraperitoneally) and intubation, a lateral thoracotomy was carried out to access the heart. The LAD occlusion was located between the pulmonary cone and the left atrial appendage 2–3 mm from the origin, using a 5–0 proline suture. The animals were maintained for 60 days after the surgery in a 12:12 h light–dark cycle at 24°C ± 1°C and a humidity of 60 ± 10% with *ad libitum* access to standard chow and water. Following the ligation or sham operation, the surviving rats were randomly assigned to four groups: sham group (sham, *n* = 8), model group (MI, *n* = 8), QLQX group (MI + QLQX, *n* = 8), and enalapril group (MI + enalapril, *n* = 8). All experimental procedures were approved by the Animal Care and Use Committee of Beijing University of Chinese Medicine and conformed with laboratory animal management and use regulations.

### Drug and Interventions

Qiliqiangxin capsules (A20170820) were purchased from Shijiazhuang Yiling Pharmaceutical Co., Ltd. (Shijiazhuang, Hebei, China). The high reproducibility of QLQX was clarified by a fingerprint analysis, which exhibited a high similarity of 10 batches ranging from 0.978 to 1.000 (Zhang J. et al., 2013). Previous chemical analysis of QLQX, which was obtained from the same supplier and had the same batch number with QLQX in this study, identified the profile and concentrations of stable active ingredients (Fan et al., 2019). Enalapril was obtained from Yangzijiang Pharmaceutical Group Jiangsu Pharmaceutical Co., Ltd. (Taizhou, Jiangsu, China) as the positive control. Both the drug powder of Qiliqiangxin capsules and enalapril were dissolved in distilled water. From the second day after the surgery, Qiliqiangxin capsules and enalapril were administered for 60 days at doses of 0.514 g/(kg. d) and 2.857 mg/(kg. d), respectively (equivalent to 10 times the clinical dose), by gavage once a day. An equal volume of distilled water was administered to the sham and MI groups.

### Echocardiography

Transthoracic echocardiography was performed non-invasively 60 days after the surgery to assess LV structure and function using a Vevo 2100 high-resolution imaging system equipped with a 15-MHz transducer (VisualSonics, Toronto, Canada). Two-dimensional, M-mode parasternal echocardiograms from the long-axis view were captured for determining the ejection fraction (EF), fractional shortening (FS), LV end-systolic, and end-diastolic internal diameters (LVIDs and LVIDd), LV end-systolic and end-diastolic volumes (LVESV and LVEDV), LV end-systolic and end-diastolic anterior wall thicknesses (LVAWs and LVAWd), and LV end-systolic and end-diastolic posterior wall thicknesses (LVPWs and LVPWd).

### Positron Emission Tomography

Regional myocardial utilization of glucose was determined by ^18^F-fluorodeoxyglucose (^18^F-FDG)-PET using a Micro PET/computed tomography (CT) scanner (Inveon, Siemens, TN, United States). ^18^F-FDG, a glucose analog radiotracer (radiochemical purity > 95%), was synthesized by HTA Co., Ltd. (Beijing, China). Following fasting for 15–19 h prior to imaging, the rats were subjected to FDG administration as a bolus intravenous injection via the tail vein with the mean activities of approximately 1.0 mCi. All *in vivo* imaging experiments were carried out under inhalation anesthesia using isoflurane (induced with 4% and maintained with 1.5–2% concentration). The animal was then positioned prone on the scanner bed with the heart centered in the field of view. A 10-min separate CT acquisition was performed for anatomical reference before PET scanning at 80 kV, 500 μA, and 260-ms exposure time with a thickness of 0.22 mm. Then, a static 15-min PET scan was initiated 60 min after FDG injection. The CT data were reconstructed using a cone beam Feldkamp reconstruction algorithm (COBRA) method, and PET images were reconstructed using a three-dimensional ordered subset expectation maximization (OSEM3D)/maximum *a posteriori* (MAP) algorithm (2/18 iterations) with the matrix size of 128 × 128 voxels and the requested resolution of 1.5 mm. The ^18^F-FDG uptake of LV myocardial segments was analyzed in the Inveon Research Workplace and quantified as a standardized uptake value (SUV). The PET images were re-angulated to typical heart short-axis and horizontal and vertical long-axis views and the regions of interest (ROI) were defined to the infarcted border area and the remote area (Tao et al., 2015; Zhang et al., 2018).

### Western Blot Analysis

Myocardial tissues in both the infarcted border area and remote area were homogenized in radio immunoprecipitation assay (RIPA) lysis buffer (Applygen Technologies, Beijing, China) for total cell protein extraction, followed by protein quantification using a bicinchoninic acid (BCA) protein assay kit (Applygen Technologies, Beijing, China). Equal amounts of proteins (50 μg) were separated on a 4–12% gradient sodium dodecyl sulfate polyacrylamide gel electrophoresis (SDS-PAGE) gel and transferred onto nitrocellulose membranes in the transfer buffer. The membranes were probed with primary antibodies diluted in 5% w/v non-fat milk/Tris-buffered saline-Tween overnight at 4°C and then incubated for 1 h with appropriate horseradish peroxidase (HRP)-conjugated secondary antibodies (diluted 1:5000, Boster Biological Technology, Wuhan, China) at room temperature. The primary antibodies against glucose transporter 4 (GLUT4, 1:2000 dilution), glucose transporter 1 (GLUT1, 1:10,000 dilution), pyruvate dehydrogenase (PDH, 1:5000 dilution), phosphorylated PDH (p-PDH, 1:5000 dilution), pyruvate dehydrogenase kinase 4 (PDK4, 1:2000 dilution), lactate dehydrogenase A (LDHA, 1:1000 dilution), fatty acid translocase (FAT/CD36, 1:500 dilution), and carnitine palmitoyl transferase-1 (CPT-1, 1:500 dilution) were all from Abcam (Cambridge, United Kingdom). Glyceraldehyde-3-phosphate dehydrogenase (GAPDH) (1:5000 dilution, Applygen Technologies) was measured as a loading control. Finally, proteins were visualized with an enhanced chemiluminescence kit (Beyotime Biotechnology, Jiangsu, China) and quantified using ImageJ.

### Histopathologic Staining

Cardiac tissue specimens embedded in paraffin were cut into 3-μm-thick transverse sections, mounted on glass slides and stained with hematoxylin and eosin (H&E) to assess cardiomyocyte morphology. Immunohistochemical analysis and immunofluorescence microscopy were also performed. Immunohistochemical staining was performed to determine several important metabolic proteins in the border and remote areas. The slides were submerged in citrate solution (pH 6.0) for heat-mediated antigen retrieval and incubated with 3% hydrogen peroxide (H_2_O_2_) to block endogenous peroxidase. After blocking with non-immune goat serum, the slices were incubated with primary antibodies raised against GLUT1 (diluted 1:500), GLUT4 (diluted 1:800), LDHA (diluted 1:1500), and CD36 (diluted 1:200) overnight at 4°C, followed by incubation with an HRP-conjugated goat anti-rabbit immunoglobulin G (IgG) secondary antibody at 37°C for 30 min. The staining was then developed with a diaminobenzidine (DAB) detection kit (ZSGB-BIO, Beijing, China). Immunofluorescence staining was carried out to observe the subcellular localization of GLUT4 and GLUT1. After permeabilization with 0.1% Triton X-100 in phosphate-buffered saline (PBS), the specimens were stained with primary antibodies against GLUT4 (diluted 1:500) and GLUT1 (diluted 1:500) overnight at 4°C and a subsequent Dylight 649-conjugated goat anti-rabbit IgG secondary antibody (Abbkine Scientific Co., Ltd., Wuhan, China) at a dilution of 1:500 for 1 h at room temperature. The slides were mounted using a fluorescent mounting medium containing 4,6-diamidino-2-phenylindole (DAPI), and the images were acquired with a Leica TCS SP8X confocal microscope (Leica, Wetzlar, Germany). Leica confocal software was employed to quantify the fluorescence intensity of sarcolemma and cytosol. The results were presented as the ratio of the expression levels of GLUTs.

### Determination of Lactate, Na^+^/K^+^-ATPase, and Ca^2+^-ATPase

The lactate content and the activities of Na^+^/K^+^-ATPase and Ca^2+^-ATPase in the border-area myocardium were determined with a lactate assay kit, an Na^+^/K^+^-ATPase assay kit, and a Ca^2+^-ATPase assay kit, respectively (Nanjing Jiancheng Bioengineering Institute, Nanjing, China). The procedures were conducted in accordance with the kit instructions strictly.

### Statistical Analysis

All data were presented as the means ± standard deviation (SD). Overall variables with normal distribution were analyzed using the Shapiro–Wilk test of normality. A one-way analysis of variance was conducted for comparisons of multiple groups, followed by pairwise comparisons with the least significant difference *post-hoc* testing or with Tamhane’ T2 otherwise. The difference between mean SUVs in the border and remote areas was analyzed using a paired *t*-test. Analyses were performed with IBM SPSS Statistics 20. A *P*-value less than 0.05 was considered statistically significant.

## Results

### Qiliqiangxin Improved Cardiac Function and Structure in Rats With HF

**FIGURE 1:**
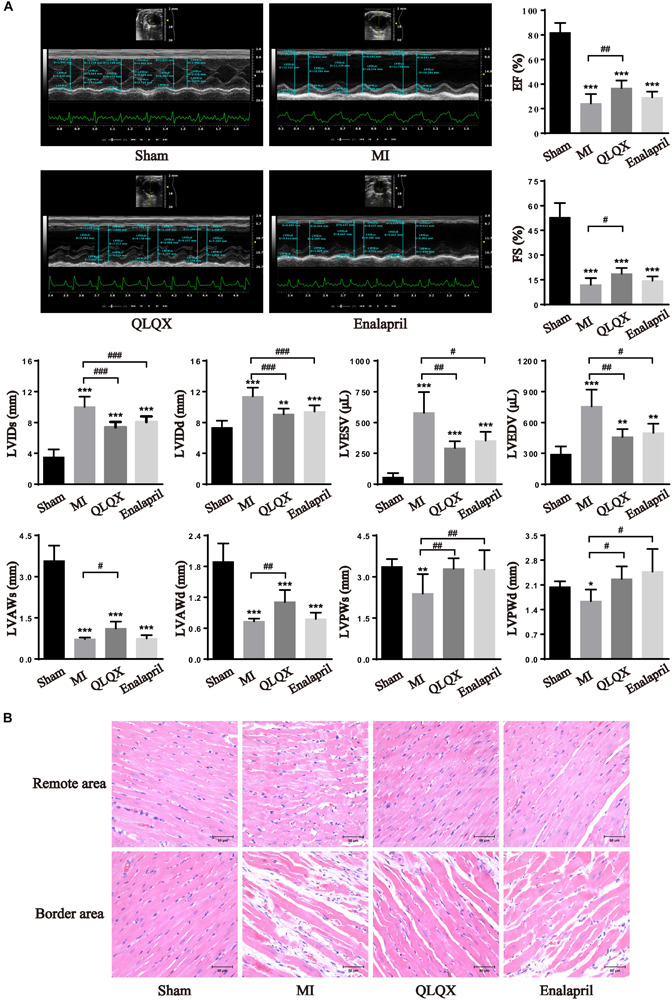
QLQX improved cardiac structure and function 8 weeks after MI. **(A)** Echocardiography parameters in all groups (*n* = 8 per group). EF: sham: 81.85 ± 7.90%; MI: 24.21 ± 7.68%; QLQX: 36.73 ± 6.16%; enalapril: 29.16 ± 4.65%. FS: sham: 53.00 ± 8.71%; MI: 12.00 ± 4.01%; QLQX: 18.65 ± 3.49%; enalapril: 14.45 ± 2.55%. LVIDs: sham: 3.52 ± 0.98 mm; MI: 10.04 ± 1.31 mm; QLQX: 7.40 ± 0.69 mm; enalapril: 8.07 ± 0.73 mm. LVIDd: sham: 7.37 ± 0.88 mm; MI: 11.39 ± 1.14 mm; QLQX: 9.09 ± 0.71 mm; enalapril: 9.44 ± 0.80 mm. LVESV: sham: 57.11 ± 33.07 μL; MI: 580.07 ± 167.32 μL; QLQX: 292.39 ± 57.34 μL; enalapril: 354.87 ± 70.37 μL. LVEDV: sham: 291.60 ± 74.64 μL; MI: 757.30 ± 162.17 μL; QLQX: 464.14 ± 75.98 μL; enalapril: 500.73 ± 89.07 μL. LVAWs: sham: 3.58 ± 0.55 mm; MI: 0.72 ± 0.05 mm; QLQX: 1.12 ± 0.25 mm; enalapril: 0.74 ± 0.13 mm. LVAWd: sham: 1.89 ± 0.35 mm; MI: 0.73 ± 0.05 mm; QLQX: 1.11 ± 0.23 mm; enalapril: 0.78 ± 0.12 mm. LVPWs: sham: 3.37 ± 0.27 mm; MI: 2.39 ± 0.70 mm; QLQX: 3.29 ± 0.38 mm; enalapril: 3.27 ± 0.71 mm. LVPWd: sham: 2.04 ± 0.16 mm; MI: 1.64 ± 0.33 mm; QLQX: 2.27 ± 0.36 mm; enalapril: 2.48 ± 0.64 mm. **(B)** H&E staining of heart tissues in the remote area and border area. Values are means ± SD. *P < 0.05, **P < 0.01, ***P < 0.001 vs. the sham group; ^#^*P* < 0.05, ^##^*P* < 0.01, ^###^*P* < 0.001 vs. the MI group.

The cardiac function and structural changes in HF rats were measured with echocardiography and H&E staining. Significant decreases in EF and FS were noted in rats with MI 8 weeks after infarction, accompanied by much higher LVIDs, LVIDd, LVESV, and LVEDV as well as lower LVAWs, LVAWd, LVPWs, and LVPWd. In response to drug treatment, QLQX ameliorated impaired EF and FS post-MI and prevented LV remodeling by reducing the diameters and volumes. Enalapril mainly improved cavity dilatation but had minimal effect on the values of EF and FS. Besides, QLQX recovered both thinned LV anterior wall and posterior wall induced by MI, but enalapril only thickened the posterior wall (Figure 1A). Consistently, the morphological assessment showed that the MI group was characterized by a moderate disarrangement in the remote-area myocardium, which was rescued by both QLQX and enalapril. Also, marked necrosis of myofibrils, inflammatory infiltration, and interstitial fibrosis in the border area was relieved by only QLQX (Figure 1B).

### Qiliqiangxin Optimized the Glucose Metabolism in the Remote- and Border-Area Myocardia in Rats With HF

**FIGURE 2:**
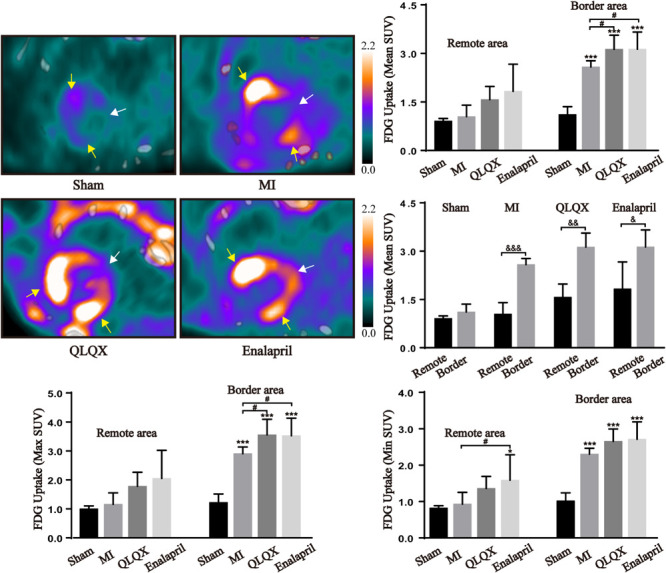
QLQX optimized the cardiac glucose metabolism 8 weeks after MI. The representative cardiac images of ^18^F-FDG PET/CT in all groups (remote area: white arrows; border area: yellow arrows) and the mean SUV, max SUV, and min SUV of ^18^F-FDG in the remote area (sham: 0.91 ± 0.08, 1.00 ± 0.10, and 0.82 ± 0.06, respectively, MI: 1.04 ± 0.36, 1.16 ± 0.40, and 0.93 ± 0.32, respectively, QLQX: 1.57 ± 0.41, 1.78 ± 0.48, and 1.36 ± 0.33, respectively, enalapril: 1.83 ± 0.84, 2.05 ± 0.97, and 1.58 ± 0.70, respectively) and in the border area (sham: 1.11 ± 0.25, 1.22 ± 0.29, and 1.02 ± 0.22, respectively, MI: 2.58 ± 0.19, 2.91 ± 0.23, and 2.30 ± 0.16, respectively, QLQX: 3.13 ± 0.44, 3.55 ± 0.54, and 2.65 ± 0.34, respectively, enalapril: 3.13 ± 0.53, 3.52 ± 0.61, and 2.71 ± 0.48, respectively) myocardium (*n* = 5 per group). Values are means ± SD. *P < 0.05, ***P < 0.001 vs. the sham group; ^#^*P* < 0.05 vs. the MI group; ^&^*P* < 0.05, ^&&^*P* < 0.01, ^&&&^*P* < 0.001 vs. the remote area.

Glucose metabolism in the myocardium was assessed using ^18^F-FDG PET/CT. The fasted sham rats showed a minimal cardiac ^18^F-FDG uptake in the remote and border areas. In comparison, a substantial uptake of ^18^F-FDG in the border area and a slight increase in the remote area were induced after MI. In the border area, a rather higher ^18^F-FDG uptake than that in rats with MI was observed in the QLQX and enalapril groups according to the mean and maximum (max) SUV with a consistent trend followed in minimum (min) SUV. However, in the remote area, the ^18^F-FDG uptake was moderately elevated by QLQX, and enalapril might exert a more obvious promoting effect primarily indicated in min SUV. The comparisons of mean SUV between the two areas in each group showed no regional difference in the sham groups, but the ^18^F-FDG uptake in the border area was much higher than that in the remote area in the MI, QLQX, and enalapril groups (Figure 2).

### Qiliqiangxin Regulated GLUT1 and GLUT4 in the Remote- and Border-Area Myocardia in Rats With HF

**FIGURE 3:**
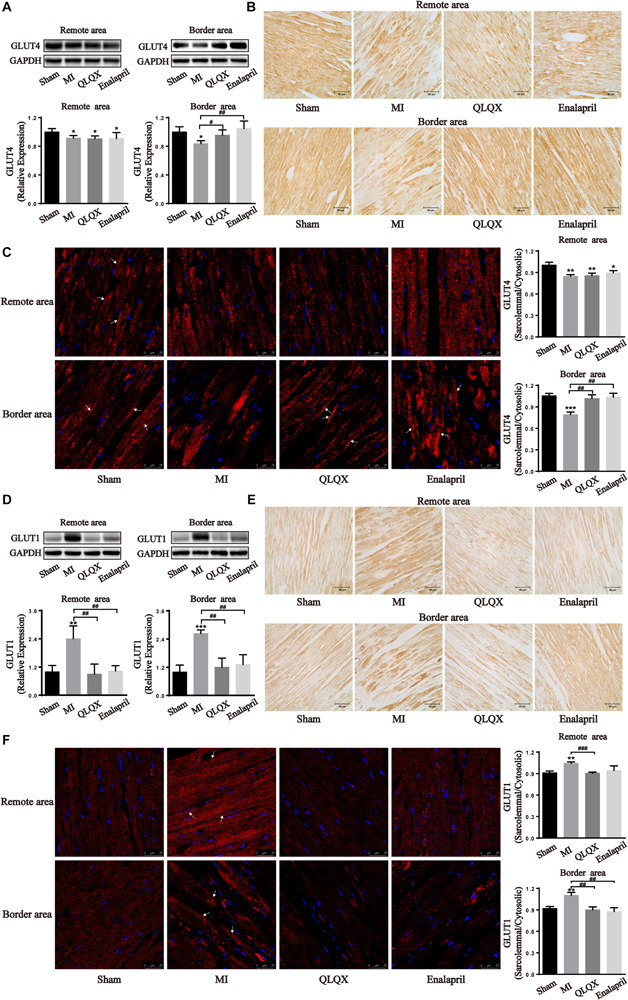
Effects of QLQX on glucose uptake transporters in infarcted hearts. **(A,D)** Western blot analysis of GLUT4 and GLUT1 protein expression in the remote area and border area (*n* = 3 or 4 per group). GLUT4: remote area: sham: 1.00 ± 0.04, MI: 0.91 ± 0.03, QLQX: 0.91 ± 0.04, enalapril: 0.91 ± 0.09; border area: sham: 1.00 ± 0.07, MI: 0.84 ± 0.04, QLQX: 0.96 ± 0.07, enalapril: 1.05 ± 0.11. GLUT1: remote area: sham: 1.00 ± 0.27, MI: 2.40 ± 0.55, QLQX: 0.90 ± 0.42, enalapril: 1.03 ± 0.23; border area: sham: 1.00 ± 0.29, MI: 2.64 ± 0.15, QLQX: 1.20 ± 0.39, enalapril: 1.32 ± 0.42. **(B,E)** Immunochemical staining of GLUT4 and GLUT1 in the remote and border areas. **(C,F)** Ratio of sarcolemmal to cytosolic expression of GLUT4 and GLUT1 and respective images of immunofluorescence confocal microscopy (indicated with arrows) in remote and border areas (*n* = 4 per group). GLUT4: remote area: sham: 1.00 ± 0.04, MI: 0.85 ± 0.02, QLQX: 0.86 ± 0.03, enalapril: 0.89 ± 0.03; border area: sham: 1.06 ± 0.03, MI: 0.79 ± 0.04, QLQX: 1.02 ± 0.05, enalapril: 1.04 ± 0.05. GLUT1: remote area: sham: 0.91 ± 0.02, MI: 1.04 ± 0.02, QLQX: 0.91 ± 0.01, enalapril: 0.94 ± 0.06; border area: sham: 0.92 ± 0.03, MI: 1.10 ± 0.04, QLQX: 0.90 ± 0.04, enalapril: 0.88 ± 0.05. **(B,E)** Immunochemical staining of GLUT4 and GLUT1 in the remote and border areas. Values are means ± SD. *P < 0.05, **P < 0.01, ***P < 0.001 vs. the sham group; ^#^*P* < 0.05, ^##^*P* < 0.01, ^###^*P* < 0.001 vs. the MI group.

To further verify metabolic changes shown in the results of ^18^F-FDG PET, the total cell protein expression levels of GLUT4 and GLUT1 contributing to glucose uptake in the heart were determined by Western blot and immunohistochemical analyses. The results showed that MI led to a significant attenuation of insulin-sensitive GLUT4 but augmentation of insulin-insensitive GLUT1 in the border and remote areas. Following the treatment with QLQX and enalapril, the protein content of GLUT4 remained unchanged in the remote area but increased in the border area (Figures 3A,B), concomitant with a great suppressive effect on GLUT1 in both areas (Figures 3D,E). Subsequently, trafficking of the two glucose transporter isoforms was detected by immunofluorescence staining. The ratio of sarcolemmal and cytosolic GLUT4 in either remote or border area was dampened by MI; the ratio in the border myocardium showed significant increments in the treated groups (Figure 3C). However, MI stimulated the ratio of GLUT1 localized in the sarcolemma to that in the cytoplasm in both areas, which obviously reduced in response to QLQX and enalapril, but had no significant effect in the enalapril group in the remote area (Figure 3F).

### Qiliqiangxin Affected the Glucose Oxidation Proteins in the Remote- and Border-Area Myocardia in Rats With HF

**FIGURE 4:**
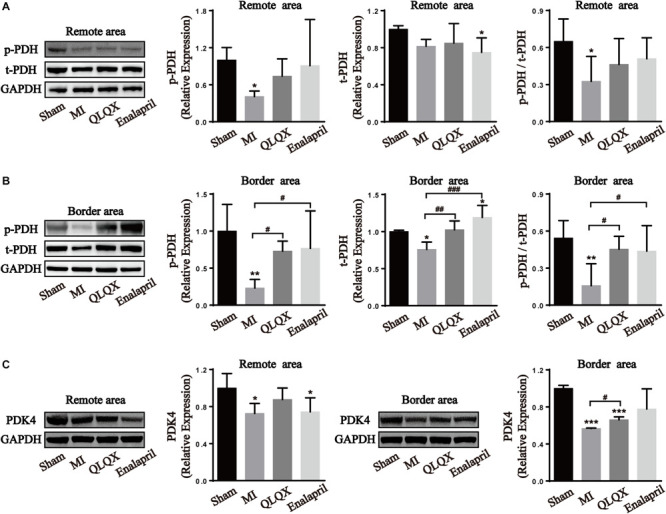
Effects of QLQX on glucose oxidation enzymes in infarcted hearts. **(A)** Cardiac level of p-PDH, t-PDH, and the ratio of p-PDH to t-PDH in the remote area (*n* = 4 per group). Sham: 1.00 ± 0.20, 1.00 ± 0.04, and 0.65 ± 0.18, respectively, MI: 0.41 ± 0.09, 0.82 ± 0.07, and 0.33 ± 0.20, respectively, QLQX: 0.74 ± 0.28, 0.85 ± 0.21, and 0.46 ± 0.21, respectively, enalapril: 0.91 ± 0.75, 0.75 ± 0.16, and 0.51 ± 0.17, respectively. **(B)** Cardiac level of p-PDH, t-PDH, and the ratio of p-PDH to t-PDH in the border area (*n* = 4 per group). Sham: 1.00 ± 0.36, 1.00 ± 0.02, and 0.54 ± 0.14, respectively, MI: 0.23 ± 0.12, 0.76 ± 0.10, and 0.16 ± 0.18, respectively, QLQX: 0.73 ± 0.14, 1.02 ± 0.12, and 0.45 ± 0.10, respectively, enalapril: 0.77 ± 0.50, 1.19 ± 0.16, and 0.44 ± 0.21, respectively. **(C)** Western blot analysis of PDK4 in remote and border areas (*n* = 4 per group). Remote area: sham: 1.00 ± 0.16; MI: 0.73 ± 0.11; QLQX: 0.88 ± 0.12; enalapril: 0.74 ± 0.15. Border area: sham: 1.00 ± 0.03; MI: 0.57 ± 0.01; QLQX: 0.66 ± 0.03; enalapril: 0.78 ± 0.22. Values are means ± SD. *P < 0.05, **P < 0.01, ***P < 0.001 vs. the sham group;^ #^*P* < 0.05, ^##^*P* < 0.01, ^###^*P* < 0.001 vs. the MI group.

PDH, the rate-limiting enzyme of glucose oxidation, is inactivated via phosphorylation by specific PDH kinase (Stanley et al., 2005). Western blot analysis of the total-PDH (t-PDH) and phospho-PDH protein (p-PDH) expression in cardiac tissues helped in figuring out the glucose oxidation changes in all groups. Decreased phosphorylation of PDH, t-PDH expression (despite no statistical difference in the remote area), and ratio of p-PDH/t-PDH in both areas were found in the MI group. However, only the reduction of phospho-PDH, t-PDH, and p-PDH/t-PDH ratio in the border area rather than in the remote area was upregulated by QLQX and enalapril (Figures 4A,B). Consistently, cardiac expression of PDK4, the predominant PDH kinase isoform in the heart, was significantly suppressed in the MI group in both areas, and treatment with QLQX induced PDK4 only in the border area (Figure 4C).

### Qiliqiangxin Modulated LDHA Expression in the Remote- and Border-Area Myocardia in Rats With HF

**FIGURE 5:**
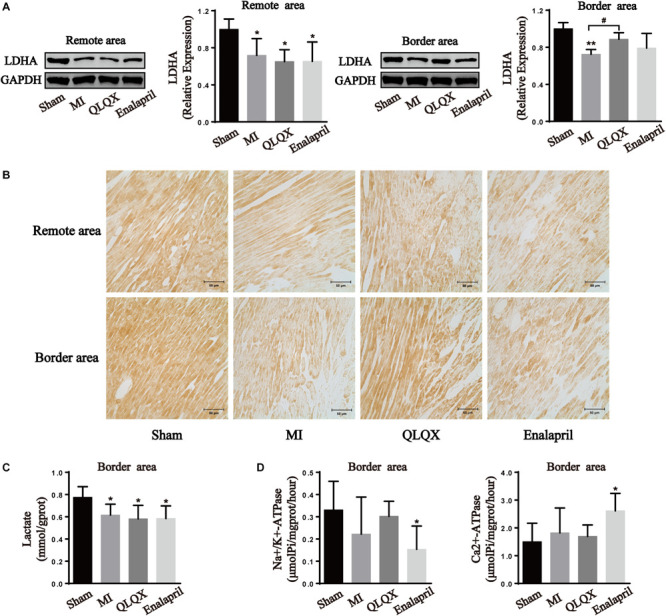
Effects of QLQX on anaerobic glycolytic enzymes in infarcted hearts. **(A)** Western blot analysis of LDHA protein expression in remote and border areas (*n* = 4 per group). Remote area: sham: 1.00 ± 0.11; MI: 0.72 ± 0.18; QLQX: 0.65 ± 0.13; enalapril: 0.65 ± 0.21. Border area: sham: 1.00 ± 0.07; MI: 0.72 ± 0.05; QLQX: 0.89 ± 0.07; enalapril: 0.79 ± 0.16. **(B)** Immunochemical staining of LDHA in the remote and border areas. **(C)** Contents of lactate in the border area (*n* = 6–8 per group). Sham: 0.78 ± 0.10; MI: 0.62 ± 0.10; QLQX: 0.58 ± 0.12; enalapril: 0.58 ± 0.11. **(D)** Activity of Na^+^/K^+^-ATPase and Ca^2+^-ATPase in the border area (*n* = 6–8 per group). Na^+^/K^+^-ATPase: sham: 0.33 ± 0.13; MI: 0.22 ± 0.17; QLQX: 0.30 ± 0.07; enalapril: 0.15 ± 0.10. Ca^2+^-ATPase: sham: 1.50 ± 0.67; MI: 1.82 ± 0.89; QLQX: 1.70 ± 0.41; enalapril: 2.61 ± 0.63. Values are means ± SD. *P < 0.05, **P < 0.01 vs. the sham group; ^#^*P* < 0.05 vs. the MI group.

Western blot and immunochemistry examinations of LDHA were performed to further determine the glycolytic process in all groups. The results show that infarcted hearts were characterized by a marked decline in LDHA protein expression in the remote and border areas. LDHA content was induced in the border area but remained unchanged in the remote area by QLQX treatment. Meanwhile, LDHA in neither the border area nor in the remote area was influenced by enalapril (Figures 5A,B).

### Qiliqiangxin Did Not Adversely Affect Lactate Production and Ionic Homeostasis in the Border-Area Myocardium in Rats With HF

The results show that the content of lactate in the border-area cardiac tissue decreased in the MI group, but QLQX and enalapril had no effect on the content (Figure 5C). A downward trend was noted for Na^+^/K^+^-ATPase of the infarcted heart. QLQX did not show obvious effects on the content, whereas the Na^+^/K^+^-ATPase activity was significantly lower in the enalapril group than in the sham rats. Meanwhile, no significant differences were observed in the Ca^2+^-ATPase of the sham, MI, and QLQX groups, and enalapril elevated cardiac Ca^2+^-ATPase activity exceeding that in the sham group (Figures 5C,D).

### Qiliqiangxin Influenced FA Metabolism Proteins in the Remote- and Border-Area Myocardia in Rats With HF

**FIGURE 6:**
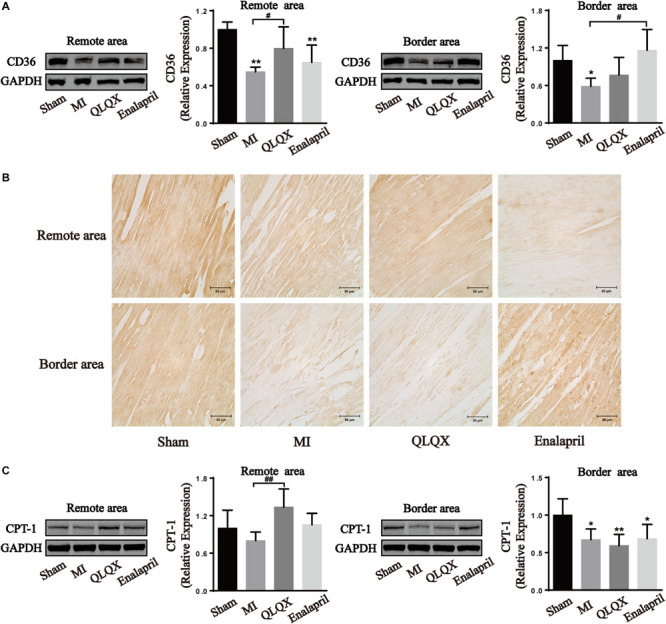
The effects of QLQX on fatty acid uptake and oxidation proteins in failing infarcted hearts. **(A)** Western blot analysis of CD36 protein expression in remote and border areas (*n* = 4 per group). Remote area: sham: 1.00 ± 0.08; MI: 0.55 ± 0.05; QLQX: 0.80 ± 0.23; enalapril: 0.65 ± 0.18. Border area: sham: 1.00 ± 0.24; MI: 0.59 ± 0.13; QLQX: 0.77 ± 0.28; enalapril: 1.16 ± 0.33. **(B)** Immunochemical staining of CD36 in remote and border areas. **(C)** Western blot analysis of CPT-1 in remote and border areas (*n* = 4 per group). Remote area: sham: 1.00 ± 0.29; MI: 0.80 ± 0.14; QLQX: 1.34 ± 0.29; enalapril: 1.06 ± 0.18. Border area: sham: 1.00 ± 0.22; MI: 0.67 ± 0.14; QLQX: 0.59 ± 0.15; enalapril: 0.69 ± 0.19. Values are means ± SD. *P < 0.05, **P < 0.01 vs. the sham group; ^#^*P* < 0.05, ^##^*P* < 0.01 vs. the MI group.

Moreover, the two proteins promoting FA metabolism were also determined. The infarcted heart showed a great reduction in the protein expression of CD36, a marker of FA uptake, and CPT-1, a marker of FA mitochondrial oxidation. QLQX significantly increased the expression level of CD36 and CPT-1 in the remote-area myocardium, but no difference between QLQX group and MI group was detectable in the border area. Enalapril appeared to upregulate only the CD36 expression in the border area (Figure 6).

## Discussion

Myocardial metabolic deterioration is the intrinsic hallmark of ischemic heart disease and HF, supporting the intriguing therapeutic perspectives of metabolic modulators (Doehner et al., 2014). However, the current pharmaceutical interventions aiming at energy substrates are worth further discussion considering their preference for a specific fuel at the expense of metabolic flexibility (Peterzan et al., 2017). Flexibility modulation is believed to be a more accurate target despite barely matched therapy. After MI, the injured ventricle undergoes a spatial heterogeneity in structural, mechanical, and metabolic remodeling response to a pathological environment (Sharov et al., 1997; Torres et al., 2018; van Duijvenboden et al., 2019). Hence, compelling distinct zones preferring the same substrate seem to compromise the substrate flexibility. It may be better to treat the infarcted heart by separating it into two sections: the border area and the remote area.

QLQX has been proven to have a great potential as a promising metabolic modulator as it can regulate cardiac glucose and FA metabolism simultaneously by upregulating a large number of enzymes, including GLUT4, GLUT1, CD36, and so forth in different pathological models (Shen et al., 2017; Wang et al., 2017, 2018). This multi-targeting regulation might depend on the multiple components identified in QLQX, encompassing ginsenoside Rb1, astragaloside, salvianolic acid A, and so forth (Zhang et al., 2019). Most of these components have demonstrated an ability to target energy metabolism (Dong et al., 2017; Zheng et al., 2017; Cui et al., 2018). Enalapril is a classic medication for HF, which blocks the renin–angiotensin–aldosterone system (Yusuf et al., 1991). It is widely believed that enalapril predominantly aims at alleviating ventricular remodeling. However, the fact that enalapril lessens cardiac workload and energy demands provides indirect evidence for its metabolism-modulating properties (Gupta and Houston, 2017). Recently, angiotensin II has been clarified to induce a pronounced elevation in the levels of GLUT1 and a suppression of FAO enzymes, CD36, CPT-1, and so forth (Pellieux et al., 2006, 2009), directly highlighting that enalapril might also be a metabolic regulator. So, with enalapril as the control intervention, the results further reveal that QLQX enabled border and remote cardiomyocytes to metabolize different substrates according to the pathological conditions to fulfill energy demands in both areas and ultimately improve LV dysfunction and LV remodeling throughout the heart.

### QLQX Protected Global LV Function and Structure

The echocardiography data from this study confirmed that a stable contractile dysfunction and LV dilatation occurred in the rat model of MI induced by LAD ligation for 8 weeks, consistent with previous results (Tao et al., 2015). QLQX could profoundly elevate the LV function. Both QLQX and enalapril alleviated cardiac dilatation (LVIDs, LVIDd, LVESV, and LVEDV). In the infarcted heart, LVAW and LVPW reduced, indicating an ongoing loss of viable myocardium, which originated from LVAW and progressively extended to LVPW. Both LVAW and LVPW were elevated by QLQX, whereas only LVPW was enhanced by enalapril. LVAW anatomically resembles the infarct and border areas, and LVPW equals the remote area. The results demonstrate that the myocardium in the border area was potentially salvageable and not only cells in the distal region, QLQX was also confirmed to preserve the survival cardiomyocytes closet to the scar. The morphological assessments support this conclusion.

### QLQX Augmented Myocardial Glucose Uptake in the Border Area

An infarcted heart is commonly assumed to undergo a shift from FAO to glucose metabolism. Animal micro PET/CT has helped obtain the region-specific properties of myocardial glucose uptake. In line with other studies (Rosenblatt-Velin et al., 2001; Zhang et al., 2018), it has been observed that MI aroused a striking increase in glucose uptake, primarily distributed in the border area. QLQX and enalapril predominately evoked a stronger glucose entry into border cardiomyocytes but only exerted moderate effects on remote cells. The comparison of the mean SUV in the border and remote areas provided evidence that QLQX treatment favored regional differences in glucose uptake. The aforementioned glucose uptake alternations might be related to the regulation of glucose transporters. The two isoforms in human hearts, GLUT4 and GLUT1, facilitate glucose transport into cardiomyocytes by trafficking from the cytoplasm to the sarcolemma (Zhang L. et al., 2013). In the present study, GLUT4 protein expression appeared lower, whereas GLUT1 expression markedly increased throughout the infarcted heart. In addition, the ratio of sarcolemmal to cytosolic contents of GLUT4 also significantly decreased in both areas, suggesting a weakened translocation. However, the trafficking of GLUT1 was notably stimulated, indicating that MI led to a glucose uptake increase, yet in a GLUT1-dependent manner. GLUT4 is insulin dependent and serves as the major isoform in the adult myocardium, and the insulin-insensitive GLUT1 predominates in fetal hearts (Tran and Wang, 2019). The transition from GLUT4 to GLUT1 has been found in ischemic and failed myocardia previously; nevertheless, the mechanism remains unknown (Remondino et al., 2000; Rosenblatt-Velin et al., 2001). In the context of HF, insulin resistance weakens GLUT4 availability (Karwi et al., 2019). Therefore, GLUT1 expression may increase to compensate. QLQX and enalapril did not modify GLUT4 in the remote area but upregulated GLUT4 content and translocation in the border area significantly. In addition, QLQX downregulated GLUT1 in the entire myocardium, and so did enalapril, probably because angiotensin II might be an underlying activator of GLUT1 (Rosenblatt-Velin et al., 2001). The observations implied that QLQX and enalapril promoted glucose uptake in the border area in a GLUT4-dependent manner.

Compared with FA, glucose is considered more oxygen effective because it consumes less oxygen to produce the same amount of ATP (Peterzan et al., 2017), making it a preferred fuel to enhance cardiac efficiency under oxygen-limited conditions. However, long-term FAO deficiency cannot be totally compensated by glucose metabolism (van Bilsen et al., 2009). Hence, the modulation of QLQX to further stimulate glucose uptake only in the border area, which is injured more severely due to oxygen deprivation, may help in protecting against hypoxic damage without excessive glucose burden in the remote area.

### QLQX Might Switch Metabolic Pathways From Glucose Oxidation to Glycolysis in the Border Area

Glucose is metabolized via oxidation under normoxic conditions alternatively via anaerobic glycolysis in oxygen deficits (Gupta and Houston, 2017). Pyruvate derived from glucose is converted into acetyl-CoA for aerobic respiration by PDH which is inhibited when phosphorylated by PDH kinases, or it is converted into lactate and toward the anaerobic glycolytic pathway by LDH (Heggermont et al., 2016). Consistent with the changes in glucose utilization shown in PET, remarkable reductions in p-PDH expression and ratio of p-PDH/t-PDH in both border and remote areas were detected as indicative of a global enhancement in the glucose oxidative process. Concomitantly, QLQX and enalapril might prevent cardiomyocytes in the border area preferring excessive glucose oxidation, but they maintained, to some extent, the augmented glucose oxidation in the remote area, which was indicated by its promoting effect on the phosphorylation of PDH only in the border area. The concordant PDK4 changes further explained the distinct modulation of PDH in both areas with QLQX.

It is expected that an uncoupling between glycolysis and glucose oxidation and augmentation of anaerobic process are induced in HF (Lopaschuk, 2017). However, the present study argues an obvious absence of LDHA in both remote and border areas indicating glycolytic dysfunction. Similar results show that cardiomyocytes in the MI border zone failed to upregulate glycolysis-related genes and revert to glycolysis resembling the neonatal metabolic phenotype (van Duijvenboden et al., 2019). Further, the activity of LDH in the myocardium declined in a 6-week MI rat model, suggesting abnormal anaerobic energy generation (Afanasiev et al., 2014). QLQX exhibited a regional effect again in that it elevated the declined LDHA expression in the border area but not in the remote area. It seems that enalapril did not play a regulatory role in LDHA expression in the two areas. The lactate content and the activity of Na^+^/K^+^-ATPase and Ca^2+^-ATPase were detected to further confirm whether QLQX could cause intracellular acidosis and disturb ion homeostasis in the border area attributed to its promoting effect on glycolysis. It became evident that the lactate production in the border myocardium of rats with MI was declined, which was consistent with the debilitated LDH expression. In a similar case, the cardiac release of purines and lactate decreased in rats with MI 8 weeks after infarction, indicating attenuated sensitivity to chronic ischemia of the infarcted myocardium (Kalkman et al., 1996). QLQX did not increase the lactate level, and the uncoupling of LDHA expression and lactate level in the QLQX group might be due to the conducive clearance of myocardial lactic acid by the active ingredients, such as ginsenoside and astragaloside (Chen et al., 2006; Yang et al., 2017). Consequently, disordered Na^+^/K^+^-ATPase and Ca^2+^-ATPase caused by intracellular acidosis was not observed in the MI and QLQX groups. Collectively, QLQX could enhance glycolysis without deteriorating lactate production and ionic homeostasis in the border area.

Taken together, it appears that QLQX drives the infarcted heart to use different glucose metabolic pathways in discrete zones via modulating key enzymes, that is, partially keeping oxidizing glucose within the remote area while transiting oxidation to anaerobic glycolysis in the border area. To save the extremely hypoxic myocytes closest to the necrosis, the shift to anaerobic metabolism may be required for rapid energy generation without using much oxygen (Abdurrachim et al., 2015). On the contrary, for the myocardium away from the scar with better perfusion, not suppressing increased glucose oxidation contributes to less accumulation of protons and lactate from glycolysis. This advantage in terms of metabolic regulation distinguishes QLQX from enalapril. Hence, it protects the myocardium in both hypoxia-injured anterior and posterior walls.

### QLQX Might Enhance FA Metabolism in the Remote Area

A dramatic decrease in FAO has been observed in HF probably due to either mitochondrial capacity abnormalities (Murphy and Hartley, 2018) or compromised FAO enzymes. In keeping with this, the present findings confirm reduced expression of CD36, a transporter involved in FA uptake, and CPT-1, which assists in FA entry into mitochondria throughout the myocardium. Further, a potential regional property of QLQX regulation on FAO was noted, which preserved the reduced CD36 and CPT-1 protein contents in the border myocytes but had a strong promoting effect on these two proteins in the remote myocardium. Reversely, enalapril only upregulated the CD36 protein expression in the border area.

FA is often discarded owing to its high oxygen consumption and potential lipotoxicity when modulating cardiac substrates in HF (Heggermont et al., 2016). However, increasing glucose by irreversibly removing FA may not be possible (He et al., 2012; Nakatani et al., 2013), and enhancing FA preference may be an alternative phenotype to restore cardiac energetics and function (Kaimoto et al., 2017). Considering that FA produces approximately three times the ATP per molecule compared with glucose (Peterzan et al., 2017), FA seems to be more efficient when oxygen is not limited in HF. Thus, stimulating FAO in the remote area may maximize energy synthesis to ensure effective functioning of myocytes. Conversely, maintaining FA utilization at a low level in the border area could avoid exacerbating hypoxic and lipotoxic injuries to the myocardium. The effects of QLQX on CD36 and CPT-1 indicate that it might exert a benign regulatory effect on cardiac FA metabolism although it requires further exploration. Enalapril has the potential to improve FA metabolism probably because angiotensin II exerts a strong suppressing effect on FAO and related genes. However, in the present study, enalapril seemed to augment only FA uptake transporter rather than the expression of FAO enzymes in the border area, implying that it was ineffective in anterior wall damage.

It is speculated that the regional metabolic regulatory advantage of QLQX might be a comprehensive outcome of its multicomponent and multi-target features. First, various components exert different regulatory effects on FAO and glycolysis. Ginsenosides, Astragaloside IV, and salsolinol, which were extracted from Ginseng Radix, Astragali Radix, and Aconiti Lateralis Radix Praeparata, in QLQX have been proven to upregulate myocardial FAO in HF (Kong et al., 2018; Tang et al., 2018; Chen et al., 2019; Wen et al., 2019). Salsolinol also could enhance glycolysis capacity, but salvianolic acid B, extracted from another herb in QLQX, inhibits cardiac glycolysis (Xu et al., 2011). It is worth noting that Astragaloside IV reduces anaerobic glycolysis in the failing heart (Dong et al., 2017), but Astragali Radix itself could increase LDH expression level in human cardiomyocytes (Wang et al., 1979), suggesting other bioactive components of Astragali Radix at work in promoting glycolysis. Second, multiple components work cooperatively. A higher salvianolic acid B concentration was found in the cardiac ischemic area than in the non-ischemic area (Xu et al., 2011). Hence, it was inferred that the distribution of other ingredients in cardiac distinct areas might also be inhomogeneous. Additionally, the regulatory effect on targets of some components also seems to have obvious regional properties in the MI heart exemplified by the effect on energy disorder induced by MI. Radix Aconiti Lateralis Preparata extract predominately optimized the distribution patterns of energy metabolism–related molecules in the non-infarct zone (Wu et al., 2019). Therefore, under different pathological conditions, the distribution of pathological targets in remote and border areas might be dissimilar, which may induce specific active ingredients to reach different regions to exert their effects.

## Conclusion

In conclusion, the observations confirmed that HF did result in a shift from FAO to glucose oxidation. For better accommodation with each microenvironment and energy demands, it is possible that QLQX could encourage the border myocardium (anterior wall) to favor anaerobic glycolysis but facilitate the remote area (posterior wall) to rely more on FA metabolism by regulating respective key enzymes, probably due to its multi-target effect. This region-dependent performance reserves metabolism flexibility to ensure energy efficiency, and a decrease in the levels of toxic intermediates in the distinct area concurrently. Despite the promising findings, the exact mechanism by which QLQX optimizes cardiac substrates regionally has yet to be determined. In addition, this study only focused on the regional issue; the metabolic modulation of QLQX in different-stage HF needs further investigation.

## Data Availability Statement

All datasets generated for this study are included in the article/supplementary material.

## Ethics Statement

The animal study was reviewed and approved by the Animal Care and Use Committee of the Beijing University of Chinese Medicine.

## Author Contributions

WC was responsible for the experiment conduction, data analyses, and manuscript writing. LW, TY, and AW assisted in performing the animal experiments. BW, TL, and ZL contributed to the PET/CT examinations and analysis. JY and YL participated in the manuscript revision. YJ, XW, and HM offered help in the immunostaining and data analysis. MZ conceived and designed this study, supervised and wrote the manuscript, and provided expertise and continuous guidance as well as necessary reagents and equipment. All authors contributed to the article and approved the submitted version.

## Conflict of Interest

The authors declare that the research was conducted in the absence of any commercial or financial relationships that could be construed as a potential conflict of interest.

## References

[B1] Abdurrachim D. Luiken J. J. Nicolay K. Glatz J. F. Prompers J. J. Nabben M. (2015). Good and bad consequences of altered fatty acid metabolism in heart failure: evidence from mouse models. *Cardiovasc. Res.* 106 194–205. 10.1093/cvr/cvv105 25765936

[B2] Afanasiev S. A. Egorova M. V. Kondratyeva D. S. Batalov R. E. Popov S. V. (2014). Comparative analysis of changes of myocardial angiogenesis and energy metabolism in postinfarction and diabetic damage of rat heart. *J. Diabetes Res.* 2014:827896. 10.1155/2014/827896 24689068 PMC3944944

[B3] Bertero E. Maack C. (2018). Metabolic remodelling in heart failure. *Nat. Rev. Cardiol.* 15 457–470. 10.1038/s41569-018-0044-6 29915254

[B4] Berthiaume J. M. Young M. E. Chen X. McElfresh T. A. Yu X. Chandler M. P. (2012). Normalizing the metabolic phenotype after myocardial infarction: impact of subchronic high fat feeding. *J. Mol. Cell Cardiol.* 53 125–133. 10.1016/j.yjmcc.2012.04.005 22542451 PMC3372615

[B5] Birkenfeld A. L. Jordan J. Dworak M. Merkel T. Burnstock G. (2019). Myocardial metabolism in heart failure: purinergic signalling and other metabolic concepts. *Pharmacol. Ther.* 194 132–144. 10.1016/j.pharmthera.2018.08.015 30149104

[B6] Chen X. Wang Q. Shao M. Ma L. Guo D. Wu Y. (2019). Ginsenoside Rb3 regulates energy metabolism and apoptosis in cardiomyocytes via activating PPARalpha pathway. *Biomed. Pharmacother.* 120:109487. 10.1016/j.biopha.2019.109487 31577975

[B7] Chen X. J. Meng D. Feng L. Bian Y. Y. Li P. Yang D. (2006). Protective effect of astragalosides on myocardial injury by isoproterenol in SD rats. *Am. J. Chin. Med.* 34 1015–1025. 10.1142/s0192415x0600448x 17163590

[B8] Cui Y. C. Yan L. Pan C. S. Hu B. H. Chang X. Fan J. Y. (2018). The contribution of different components in QiShenYiQi Pills(R) to its potential to modulate energy metabolism in protection of ischemic myocardial injury. *Front. Physiol.* 9:389. 10.3389/fphys.2018.00389 29755361 PMC5932340

[B9] Doehner W. Frenneaux M. Anker S. D. (2014). Metabolic impairment in heart failure: the myocardial and systemic perspective. *J. Am. Coll. Cardiol.* 64 1388–1400. 10.1016/j.jacc.2014.04.083 25257642

[B10] Dong Z. Zhao P. Xu M. Zhang C. Guo W. Chen H. (2017). Astragaloside IV alleviates heart failure via activating PPARalpha to switch glycolysis to fatty acid beta-oxidation. *Sci. Rep.* 7:2691. 10.1038/s41598-017-02360-5 28578382 PMC5457407

[B11] Fan C. Tang X. Ye M. Zhu G. Dai Y. Yao Z. (2019). Qi-Li-Qiang-Xin alleviates isoproterenol-induced myocardial injury by inhibiting excessive autophagy via activating AKT/mTOR pathway. *Front. Pharmacol.* 10:1329. 10.3389/fphar.2019.01329 31780944 PMC6861302

[B12] Gupta A. Houston B. (2017). A comprehensive review of the bioenergetics of fatty acid and glucose metabolism in the healthy and failing heart in nondiabetic condition. *Heart Fail Rev.* 22 825–842. 10.1007/s10741-017-9623-6 28536966

[B13] Han A. Lu Y. Zheng Q. Zhang J. Zhao Y. Zhao M. (2018). Qiliqiangxin attenuates cardiac remodeling via inhibition of TGF-beta1/Smad3 and NF-kappaB signaling pathways in a rat model of myocardial infarction. *Cell Physiol. Biochem.* 45 1797–1806. 10.1159/000487871 29510381

[B14] He L. Kim T. Long Q. Liu J. Wang P. Zhou Y. (2012). Carnitine palmitoyltransferase-1b deficiency aggravates pressure overload-induced cardiac hypertrophy caused by lipotoxicity. *Circulation* 126 1705–1716. 10.1161/circulationaha.111.075978 22932257 PMC3484985

[B15] Heggermont W. A. Papageorgiou A. P. Heymans S. van Bilsen M. (2016). Metabolic support for the heart: complementary therapy for heart failure? *Eur. J. Heart Fail.* 18 1420–1429. 10.1002/ejhf.678 27813339

[B16] Kaimoto S. Hoshino A. Ariyoshi M. Okawa Y. Tateishi S. Ono K. (2017). Activation of PPAR-alpha in the early stage of heart failure maintained myocardial function and energetics in pressure-overload heart failure. *Am. J. Physiol. Heart Circ. Physiol.* 312 H305–H313. 10.1152/ajpheart.00553.2016 28011586

[B17] Kalkman E. A. Saxena P. R. Schoemaker R. G. (1996). Sensitivity to ischemia of chronically infarcted rat hearts; effects of long-term captopril treatment. *Eur. J. Pharmacol.* 298 121–128. 10.1016/0014-2999(95)00784-9 8867098

[B18] Karwi Q. G. Zhang L. Wagg C. S. Wang W. Ghandi M. Thai D. (2019). Targeting the glucagon receptor improves cardiac function and enhances insulin sensitivity following a myocardial infarction. *Cardiovasc. Diabetol.* 18:1. 10.1186/s12933-019-0806-4 30626440 PMC6325856

[B19] Kolwicz S. C. Jr. Purohit S. Tian R. (2013). Cardiac metabolism and its interactions with contraction, growth, and survival of cardiomyocytes. *Circ. Res.* 113 603–616. 10.1161/circresaha.113.302095 23948585 PMC3845521

[B20] Kong H. L. Hou A. J. Liu N. N. Chen B. H. Dai S. N. Huang H. T. (2018). The effects of ginsenoside Rb1 on fatty acid beta-oxidation, mediated by AMPK, in the failing heart. *Iran. J. Basic Med. Sci.* 21 731–737. 10.22038/ijbms.2018.24002.6016 30140413 PMC6098964

[B21] Li X. Liu Y. Ma H. Guan Y. Cao Y. Tian Y. (2016). Enhancement of glucose metabolism via PGC-1alpha participates in the cardioprotection of chronic intermittent hypobaric hypoxia. *Front. Physiol.* 7:219. 10.3389/fphys.2016.00219 27375497 PMC4896962

[B22] Li X. Zhang J. Huang J. Ma A. Yang J. Li W. (2013). A multicenter, randomized, double-blind, parallel-group, placebo-controlled study of the effects of qili qiangxin capsules in patients with chronic heart failure. *J. Am. Coll. Cardiol.* 62 1065–1072. 10.1016/j.jacc.2013.05.035 23747768

[B23] Liang T. Zhang Y. Yin S. Gan T. An T. Zhang R. (2016). Cardio-protecteffect of qiliqiangxin capsule on left ventricular remodeling, dysfunction and apoptosis in heart failure rats after chronic myocardial infarction. *Am. J. Transl. Res.* 8 2047–2058. 27347313 PMC4891418

[B24] Liao R. Jain M. Cui L. D’Agostino J. Aiello F. Luptak I. (2002). Cardiac-specific overexpression of GLUT1 prevents the development of heart failure attributable to pressure overload in mice. *Circulation* 106 2125–2131. 10.1161/01.cir.0000034049.61181.f3 12379584

[B25] Lionetti V. Stanley W. C. Recchia F. A. (2011). Modulating fatty acid oxidation in heart failure. *Cardiovasc. Res.* 90 202–209. 10.1093/cvr/cvr038 21289012 PMC3078800

[B26] Lopaschuk G. D. (2017). Metabolic modulators in heart disease: past, present, and future. *Can. J. Cardiol.* 33 838–849. 10.1016/j.cjca.2016.12.013 28279520

[B27] Lytvyn Y. Bjornstad P. Udell J. A. Lovshin J. A. Cherney D. Z. I. (2017). Sodium glucose cotransporter-2 inhibition in heart failure: potential mechanisms, clinical applications, and summary of clinical trials. *Circulation* 136 1643–1658. 10.1161/CIRCULATIONAHA.117.030012 29061576 PMC5846470

[B28] Murphy M. P. Hartley R. C. (2018). Mitochondria as a therapeutic target for common pathologies. *Nat. Rev. Drug Discov.* 17 865–886. 10.1038/nrd.2018.174 30393373

[B29] Nakatani K. Masuda D. Oka T. Okada T. Kawase R. Nakaoka H. (2013). Pressure overload induces hypertrophy and impaired cardiac function in long-chain fatty acid transporter CD36 knockout mice. *Circulation* 128:A12900.

[B30] Noordali H. Loudon B. L. Frenneaux M. P. Madhani M. (2018). Cardiac metabolism – a promising therapeutic target for heart failure. *Pharmacol. Ther.* 182 95–114. 10.1016/j.pharmthera.2017.08.001 28821397

[B31] Pellieux C. Aasum E. Larsen T. S. Montessuit C. Papageorgiou I. Pedrazzini T. (2006). Overexpression of angiotensinogen in the myocardium induces downregulation of the fatty acid oxidation pathway. *J. Mol. Cell Cardiol.* 41 459–466. 10.1016/j.yjmcc.2006.06.004 16859699

[B32] Pellieux C. Montessuit C. Papageorgiou I. Lerch R. (2009). Angiotensin II downregulates the fatty acid oxidation pathway in adult rat cardiomyocytes via release of tumour necrosis factor-alpha. *Cardiovasc. Res.* 82 341–350. 10.1093/cvr/cvp004 19131364

[B33] Peterzan M. A. Lygate C. A. Neubauer S. Rider O. J. (2017). Metabolic remodeling in hypertrophied and failing myocardium: a review. *Am. J. Physiol. Heart. Circ. Physiol.* 313 H597–H616. 10.1152/ajpheart.00731.2016 28646030

[B34] Remondino A. Rosenblatt-Velin N. Montessuit C. Tardy I. Papageorgiou I. Dorsaz P. A. (2000). Altered expression of proteins of metabolic regulation during remodeling of the left ventricle after myocardial infarction. *J. Mol. Cell Cardiol.* 32 2025–2034. 10.1006/jmcc.2000.1234 11040106

[B35] Rosenblatt-Velin N. Montessuit C. Papageorgiou I. Terrand J. Lerch R. (2001). Postinfarction heart failure in rats is associated with upregulation of GLUT-1 and downregulation of genes of fatty acid metabolism. *Cardiovasc. Res.* 52 407–416. 10.1016/s0008-6363(01)00393-5 11738057

[B36] Sharov V. G. Sabbah H. N. Ali A. S. Shimoyama H. Lesch M. Goldstein S. (1997). Abnormalities of cardiocytes in regions bordering fibrous scars of dogs with heart failure. *Int. J. Cardiol.* 60 273–279. 10.1016/s0167-5273(97)00117-4 9261638

[B37] Shen S. Jiang H. Bei Y. Zhang J. Zhang H. Zhu H. (2017). Qiliqiangxin attenuates adverse cardiac remodeling after myocardial infarction in ovariectomized mice via activation of PPARgamma. *Cell Physiol. Biochem.* 42 876–888. 10.1159/000478641 28647730

[B38] Stanley W. C. Recchia F. A. Lopaschuk G. D. (2005). Myocardial substrate metabolism in the normal and failing heart. *Physiol. Rev.* 85 1093–1129. 10.1152/physrev.00006.2004 15987803

[B39] Tang B. Zhang J. G. Tan H. Y. Wei X. Q. (2018). Astragaloside IV inhibits ventricular remodeling and improves fatty acid utilization in rats with chronic heart failure. *Biosci. Rep.* 38:BSR20171036. 10.1042/BSR20171036 29301869 PMC6048210

[B40] Tang W. H. W. Huang Y. (2013). Cardiotonic modulation in heart failure: insights from traditional Chinese medicine. *J. Am. Coll. Cardiol.* 62 1073–1074. 10.1016/j.jacc.2013.05.028 23747774 PMC4024708

[B41] Tao L. Shen S. Fu S. Fang H. Wang X. Das S. (2015). Traditional chinese medication qiliqiangxin attenuates cardiac remodeling after acute myocardial infarction in mice. *Sci. Rep.* 5:8374. 10.1038/srep08374 25669146 PMC4648480

[B42] Torres W. M. Jacobs J. Doviak H. Barlow S. C. Zile M. R. Shazly T. (2018). Regional and temporal changes in left ventricular strain and stiffness in a porcine model of myocardial infarction. *Am. J. Physiol. Heart Circ. Physiol.* 315 H958–H967. 10.1152/ajpheart.00279.2018 30004234 PMC6230914

[B43] Tran D. H. Wang Z. V. (2019). Glucose metabolism in cardiac hypertrophy and heart failure. *J. Am. Heart Assoc.* 8:e012673. 10.1161/jaha.119.012673 31185774 PMC6645632

[B44] van Bilsen M. van Nieuwenhoven F. A. van der Vusse G. J. (2009). Metabolic remodelling of the failing heart: beneficial or detrimental? *Cardiovasc. Res.* 81 420–428. 10.1093/cvr/cvn282 18854380

[B45] van Duijvenboden K. de Bakker D. E. M. Man J. C. K. Janssen R. Gunthel M. Hill M. C. (2019). Conserved NPPB+ border zone switches from MEF2- to AP-1-driven gene program. *Circulation* 140 864–879. 10.1161/circulationaha.118.038944 31259610

[B46] Wang B. Z. Yang M. G. Pang L. Yu Z. J. (1979). [Effect of Hong-Hua (Flos Carthami) on the extent of myocardial ischemia in the different infarct zones following coronary occlusion in the dog (author’s transl)]. *Yao Xue Xue Bao* 14 474–479. 532647

[B47] Wang J. Li Z. Wang Y. Zhang J. Zhao W. Fu M. (2017). Qiliqiangxin enhances cardiac glucose metabolism and improves diastolic function in spontaneously hypertensive rats. *Evid. Based Complement Alternat. Med.* 2017:3197320. 10.1155/2017/3197320 28706558 PMC5494577

[B48] Wang Y. Han X. Fu M. Wang J. Song Y. Liu Y. (2018). Qiliqiangxin attenuates hypoxia-induced injury in primary rat cardiac microvascular endothelial cells via promoting HIF-1alpha-dependent glycolysis. *J. Cell Mol. Med.* 22 2791–2803. 10.1111/jcmm.13572 29502357 PMC5908112

[B49] Wen J. Zhang L. Liu H. Wang J. Li J. Yang Y. (2019). Salsolinol attenuates doxorubicin-induced chronic heart failure in rats and improves mitochondrial function in H9c2 cardiomyocytes. *Front. Pharmacol.* 10:1135. 10.3389/fphar.2019.01135 31680945 PMC6797600

[B50] Wolf P. Winhofer Y. Krssak M. Smajis S. Harreiter J. Kosi-Trebotic L. (2016). Suppression of plasma free fatty acids reduces myocardial lipid content and systolic function in type 2 diabetes. *Nutr. Metab. Cardiovasc. Dis.* 26 387–392. 10.1016/j.numecd.2016.03.012 27118107

[B51] Wu A. Zhai J. Zhang D. Lou L. Zhu H. Gao Y. (2013). Effect of wenxin granule on ventricular remodeling and myocardial apoptosis in rats with myocardial infarction. *Evid. Based Complement Alternat. Med.* 2013:967986. 10.1155/2013/967986 23997803 PMC3755410

[B52] Wu H. Liu X. Gao Z. Y. Dai Z. F. Lin M. Tian F. (2019). Anti-myocardial infarction effects of radix aconiti lateralis preparata extracts and their influence on small molecules in the heart using matrix-assisted laser desorption/ionization-mass spectrometry imaging. *Int. J. Mol. Sci.* 20:4837. 10.3390/ijms20194837 31569464 PMC6801437

[B53] Xu L. Deng Y. Feng L. Li D. Chen X. Ma C. (2011). Cardio-protection of salvianolic acid B through inhibition of apoptosis network. *PLoS One* 6:e24036. 10.1371/journal.pone.0024036 21915278 PMC3167815

[B54] Yan J. Young M. E. Cui L. Lopaschuk G. D. Liao R. Tian R. (2009). Increased glucose uptake and oxidation in mouse hearts prevent high fatty acid oxidation but cause cardiac dysfunction in diet-induced obesity. *Circulation* 119 2818–2828. 10.1161/circulationaha.108.832915 19451348 PMC2765220

[B55] Yang T. Lu Z. Wang L. Zhao Y. Nie B. Xu Q. (2019). Dynamic changes in brain glucose metabolism and neuronal structure in rats with heart failure. *Neuroscience* 424 34–44. 10.1016/j.neuroscience.2019.10.008 31704495

[B56] Yang Y. L. Li J. Liu K. Zhang L. Liu Q. Liu B. (2017). Ginsenoside Rg5 increases cardiomyocyte resistance to ischemic injury through regulation of mitochondrial hexokinase-II and dynamin-related protein 1. *Cell Death Dis.* 8:e2625. 10.1038/cddis.2017.43 28230856 PMC5386487

[B57] Yusuf S. Pitt B. Davis C. E. Hood W. B. Cohn J. N. (1991). Effect of enalapril on survival in patients with reduced left ventricular ejection fractions and congestive heart failure. *N. Engl. J. Med.* 325 293–302. 10.1056/nejm199108013250501 2057034

[B58] Zhang J. Wei C. Wang H. Tang S. Jia Z. Wang L. (2013). Protective effect of qiliqiangxin capsule on energy metabolism and myocardial mitochondria in pressure overload heart failure rats. *Evid. Based Complement Alternat. Med.* 2013:378298. 10.1155/2013/378298 24078824 PMC3775405

[B59] Zhang L. Jaswal J. S. Ussher J. R. Sankaralingam S. Wagg C. Zaugg M. (2013). Cardiac insulin-resistance and decreased mitochondrial energy production precede the development of systolic heart failure after pressure-overload hypertrophy. *Circ. Heart Fail.* 6 1039–1048. 10.1161/circheartfailure.112.000228 23861485

[B60] Zhang Q. Shao M. Zhang X. Wang Q. Guo D. Yang X. (2018). The effect of chinese medicine on lipid and glucose metabolism in acute myocardial infarction through PPARgamma pathway. *Front. Pharmacol.* 9:1209. 10.3389/fphar.2018.01209 30405421 PMC6207917

[B61] Zhang Y. Zhu M. Zhang F. Zhang S. Du W. Xiao X. (2019). Integrating pharmacokinetics study, network analysis, and experimental validation to uncover the mechanism of qiliqiangxin capsule against chronic heart failure. *Front. Pharmacol.* 10:1046. 10.3389/fphar.2019.01046 31619994 PMC6759796

[B62] Zheng X. Wang S. Zou X. Jing Y. Yang R. Li S. (2017). Ginsenoside Rb1 improves cardiac function and remodeling in heart failure. *Exp. Anim.* 66 217–228. 10.1538/expanim.16-0121 28367863 PMC5543242

